# Role of Physical Activity in the Prevention and Treatment of Influenza: A Review

**DOI:** 10.1186/s40798-023-00660-x

**Published:** 2023-12-02

**Authors:** Maolin You

**Affiliations:** Physical Education College, Jiangxi Normal University, No. 99, Ziyang Street, Gaoxin District, Nanchang, 330022 Jiangxi China

**Keywords:** Influenza, Physical activity, Prevention and treatment, Exercise prescription

## Abstract

**Background:**

Many concerns regarding respiratory diseases, including influenza, emerged during the epidemic of COVID-19. There were relevant research findings and suggestions for influenza prevention and treatment through physical activity, but little report about the total efficiency. So, this review was to summarize the role of physical activity in influenza prevention and treatment.

**Main Body:**

The databases Web of Science, Google Scholar, EBSCO, PubMed, CNKI, and Science Direct were used to search the related literatures. The first search ran from July to October, 2021, and the second search was conducted in September, 2023. Those publications that reported the effects of physical activity, exercise, and sport on influenza, flu, and cold were included. It found that long-term adherence to moderate physical activity is beneficial in enhancing the body’s ability to resist influenza viruses. However, high-intensity endurance physical activity can cause an open window in the human immune system, which increases the risk of infection by influenza viruses. The patients with influenza infections can participate in moderate physical activity during the pre-onset period, but some of the researchers do not recommend physical activity for patients with influenza, avoiding the transmission of influenza viruses to others through human contact of physical activity. Moreover, animal studies have shown that physical activity may worsen influenza disease. While studies found that moderate physical activity is beneficial for preventing influenza, as most experimental studies were conducted on animals, the mechanisms in human with physical activity are still unclear. No study has yet suggested exercise prescriptions to prevent and control influenza, and there is currently no way to prevent or control influenza just through physical activity. The follow-up research is needed to increase human clinical experiments, elucidate the effect of physical activity on influenza, develop exercise prescriptions and gradually promote physical activity as a practical means for preventing and treating influenza.

**Short Conclusions:**

Overall, participating in moderate physical activity regularly should be beneficial in influenza prevention, alleviating the patients’ symptoms and increasing the recovery efficiency, but this needs more testing in clinical human trials.

## Background

The Corona Virus Disease 2019 (COVID-19) outbreak has raised concerns regarding respiratory diseases, including influenza. Influenza is an acute respiratory viral infection that poses a serious threat to human lives and health and is a significant disease burden on society [1]. The World Health Organization (WHO) listed influenza among the top 10 threats to global health in 2019 [2]. An average of 88,100 additional influenza-related respiratory disease deaths are reported annually in China, corresponding to 8.2% of all deaths attributed to respiratory diseases [3]. Because it can lead to severe morbidity and mortality [4], combating influenza is an essential topic in global public health. In addition to medical treatments, researchers have also focused on Chinese medicine, dietary therapy and physical activity, which have fewer adverse effects and better prevention and control results [5]; in particular, physical activity is benefit for releasing interleukin 6 (IL-6) into the blood and benefiting from its anti-inflammatory effects [6], which can help prevent influenza infection and enable patients to recover early [7]. Consequently, physical activity is commonly recommended by researchers. The present review introduces relevant research findings and provides reference suggestions for influenza prevention and treatment through reasonable physical activity. The literature in the databases Web of Science, Google Scholar, EBSCO, PubMed, China National Knowledge Infrastructure (CNKI), and Science Direct were searched with the key words “physical activity/ exercise/ sport” + “influenza/flu/cold”. The first search ran from July to October, 2021, and the second search was conducted in September, 2023, and those studies that did not propose any specific effects or methods for influenza prevention and treatment with physical activity were excluded.

## Main Text

Physical activities help the respiratory system expel viruses, increase the concentration of white blood cells to improve immunity, raise the body temperature to help kill viruses and increase hormones to relieve psychological stress, which are important means for preventing influenza [8], so many researchers agree that physical activity can strengthen body immunity and provide a preventive effect against influenza infection [9, 10].

### Long-Term Adherence to Regular and Moderate Physical Activity Can Help Prevent Influenza Infection

**Fig. 1:**
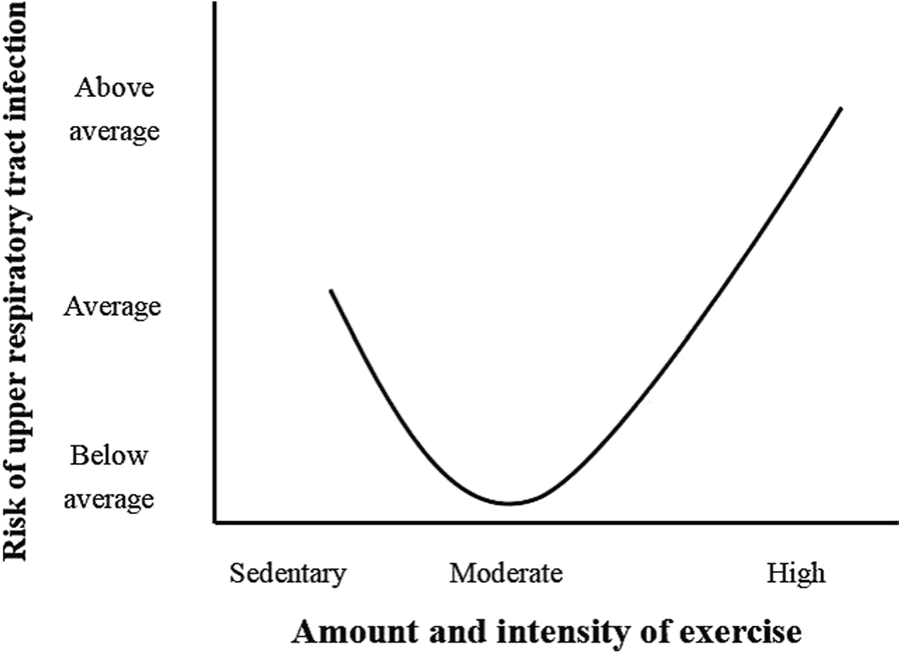
“J” shaped model of relationship between varying amounts of exercise and risk of upper respiratory tract infection (Reproduced from Nieman [16], with permission)

Regular and long-term physical activity can improve the immune system’s response and reduce the incidence of infection by the influenza viruses [11–13]. While 30 min of moderate physical activity per day can keep the body’s defense system in good condition, the intensity and duration of physical activity should not be too high, because prolonged vigorous physical activity will reduce the number of white blood cells in the body [14]. In addition, if the interval between physical activity sessions does not allow immune recovery, the risk of infection increases substantially, forming an open window for susceptibility to influenza virus infection [15]. Nieman used a “J” shaped curve to illustrate the relationship between upper respiratory tract infections and the intensity of physical activity [16], showing that moderate-to-low intensity physical activity helps protect against influenza virus infection and moderate-to-high intensity physical activity increasing the risk of infection (Fig. 1). Therefore, physical activity should not be used as an acute effort to prevent influenza and sudden moderate and higher intensity physical activity can increases the risk of influenza infection in humans who do not participate in physical activity regularly [10, 11, 17].

Since influenza epidemics pose greater threats to special populations, the results showed, firstly that physical activity helps enhance the ability of the elderly to resist influenza virus infection [18–20]. For example, Kohut, et al. divided 56 adults aged 62 years and older into three groups: active (> or = 20 min vigorous exercise three or more times per week), moderately active (regular exercise but with less intensity, frequency, and/or duration), or sedentary (no exercise). Frozen serum antibody analysis performed after 2 weeks showed higher influenza antibody levels and influenza-specific lymphocyte proliferation in the group that exercised [21]. Secondly, physical activity was more beneficial in enhancing immunity against influenza in non-obese individuals [22]. For example, Warren reported that physical activity has been shown to increase resistance to influenza infection in both obese and non-obese mice, with a more pronounced effect in non-obese mice [23]. Thirdly, physical activity improved the ability of older obese women to resist influenza viruses. Chubak, et al. conducted a 1-year moderate-intensity physical activity program for 115 overweight, obese, sedentary lifestyle, and postmenopausal women. The results showed that the intervention reduced the risk of respiratory infections in postmenopausal, nonsmoking, and overweight or obese women [24].

In summary, habitual physical activity helps the body to prevent infection by the influenza virus. This benefit is equally effective in the elderly, obese, and other populations. Therefore, it is important to actively promote fitness for all and encourage regular participation in physical activity.

### High-intensity Endurance Physical Activities are Detrimental to Influenza Prevention and Control

Epidemiological surveys have reported that moderate-to-vigorous physical activity helps people reduce influenza virus infections; for example, Siu et al. analyzed data from a health survey of 114,364 people and found that moderate-to-vigorous physical activity may reduce influenza virus infections [25]. Fondel, et al. surveyed 1509 Swedish people aged 20–60 years to assess the relationship between illness, lifestyle, and respiratory infections, reporting that higher intensity physical activity contributed to a reduced risk of upper respiratory infections [26]. Matthews, et al. conducted a 12-month follow-up of 547 healthy adults aged 20–70 years every 90 days, reporting that moderate-to-vigorous physical activity reduced the risk of upper respiratory tract infections by 20% [27].

However, the above findings have not been validated by experimental studies, which found that the stress response caused by acute, high-intensity, endurance exercise impairs the body’s immune mechanisms and reduces the efficiency of the nonspecific immune response, making organisms more susceptible to influenza virus infections. For example, one study assessed the incidence of symptoms of upper respiratory tract infections in 150 randomly selected runners participating in a 56-km run. The incidence rates were compared with those among individually matched controls who did not run. Symptoms of upper respiratory tract infections occurred in 33% of runners and in 15% of controls and were most common in those who achieved faster race times. The incidence in slow runners was no greater than that in controls [27]. Murphy, et al. divided mice into exercise and no-exercise groups. The exercise group ran on a treadmill at high intensity until becoming fatigued. Then, both groups were inoculated intranasally with a standard dose [0.25 hemagglutinating units (HAU)] of virus. The morbidity, mortality, and severity of symptoms on days 6 and 7 were significantly higher in the exercise group than in the no-exercise group [11].

Thus, some levels of physical activity may be too high for influenza immunity and may be counterproductive [10]. Although no evidence shows that low-to-moderate intensity physical activity protects humans from influenza virus infection, high-intensity physical activity clearly increases the risk of infection. Physical activity in preventing influenza should be limited to less than vigorous intensity and maintained as a daily behavior over many years.

### Uncertainty Regarding Whether Patients with Influenza Should Participate in Physical Activity

**Fig. 2:**
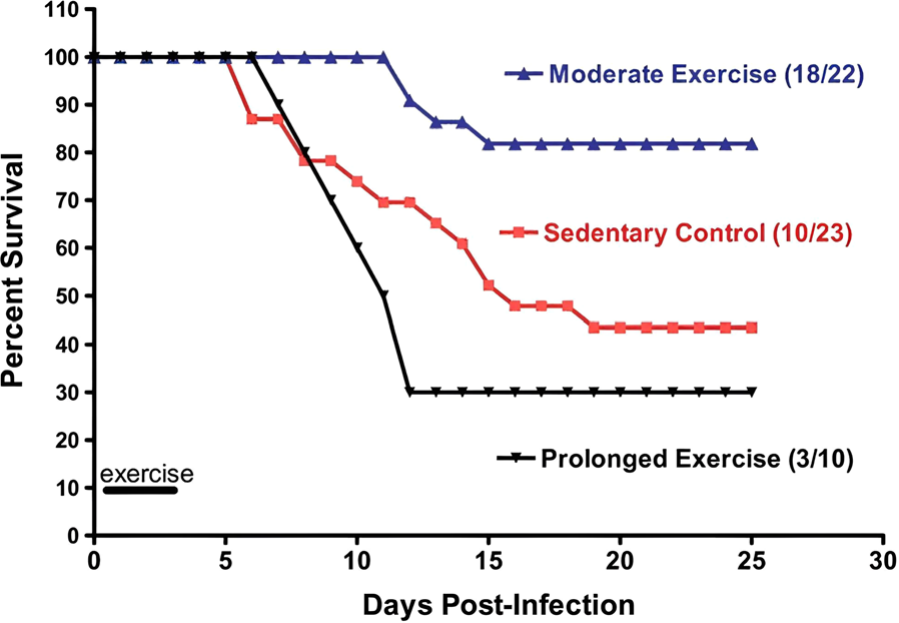
Influence of exercise on mortality due to influenza (Reproduced from Lowder et al. [34], with permission)

When bacteria and influenza viruses invade, white blood cells will firstly respond to protect the body. Physical activity can increase the number of white blood cells in blood, promoting the body mechanisms to resist the virus and repair itself. It can also enhance immune cells’ ability to recognize and kill influenza viruses [29] and reduce stress in the body, relieving anxiety and improving human resistance to influenza viruses [30]. Both human and animal studies support the practice of moderate physical activity by patients with influenza [31, 32]. For example, Klentrou, et al. implemented an intervention involving moderate physical activity for nine patients infected with influenza, in which the patients’ immunoglobulin A in salivary [IgA_s_] and the ratio [IgA_s_]: [Alb_s_] (albumin in saliva) increased and influenza symptoms were significantly relieved [33]. Furthermore, Lowder, Padgett, and Woods randomly assigned male Balb/cByJ mice to three groups: sedentary control (CON), moderate (MOD) exercise (8–12 m/min for 20–30 min), and prolonged (PRO) exercise (8–12 m/min for 2.5 h) groups. The mice ran on a treadmill 4 h after infection with influenza virus and for an additional 3 consecutive days after symptom onset. The results showed significantly reduced total cell infiltration and interferon-γ (IFN-γ) gene expression in lung tissues of mice in the MOD group, as well as a qualitative change in cytokine expression in lung tissues from type 1 T-helper (Th1) cells' response to type 2 T-helper (Th2) cells. Mice infected with influenza virus underwent early physical activity intervention, in which moderate physical activity changed their immune responses by reducing the level of Th1. The resulting altered immune response contributed to their improved survival (Fig. 2) [34]. Based on these findings, Woods recommended moderate physical activity for patients with mild upper respiratory symptoms (e.g., runny nose, sinus congestion, and sore throat) [10].

However, some researchers do not recommend physical activity for people with influenza infections because: (1) physical activity weakens the efficiency of the immune system and causes the body to lose water, which is not conducive to recovery; moreover, physical activity also imposes the risk of transmitting the influenza virus to others [35]; (2) colds cause the body to become feverish, and physical activity further raises the body temperature and places the heart under greater stress, which may lead to heart failure [36]; (3) physical activity stress increases the risk of upper respiratory tract infections [11], and this idea is corroborated by experiments. For example, Gross, et al. randomly divided eight horses into exercise and non-exercise groups: the horses in the exercise group infected with influenza virus performed training on a treadmill, while the horses in the non-exercise group were confined to their stalls. After 28 days of continuous training (the horses were trained at a speed of 6.2 m/h for 20 min on a 10° inclined treadmill 5 days/week for 28 days), all horses showed symptoms of illness (fever, cough, and mucopurulent nasal discharge), but the exercise group showed more severe symptoms [37].

There is no definitive answer to the question of whether people with influenza can participate in physical activity. However, Pedersen, Friman, and Wesslén suggested that for flu symptoms “above the neck” (stuffy or runny nose, sneezing, watery eyes, itchy throat, and no fever), light physical activity can be performed in moderation, while for symptoms “below the neck” (muscle aches, discomfort, dry cough, nausea, vomiting, diarrhea, fever), physical activity participation should be avoided [28].

## Suggestions

### Increase Human Clinical Trials

Influenza viruses differ from common cold viruses and can be life-threatening [38]. The experts warned that athletes are more vulnerable to germs than the general population and that prolonged strenuous or acute physical activity compromises the immune system, leading to increased risk of upper respiratory tract infections within a short period (e.g., less than 24 h) [39]. Therefore, practicing physical activity, especially during the broken window period of body immunity caused by excessive athletic activity, can increase the risk of influenza infection. Moreover, as exposure to infectious agents during physical activity may also increase the chance of infection, clinical trials are urgently needed to develop a scientifically evidenced physical activity program for influenza prevention. The lack of such programs may be owing to the high ethical risk of human trials, limited evidence from epidemiological surveys and animal studies, and the limited sample size and intervention validity of clinical trials, which have resulted in unconvincing conclusions. More clinical trials are needed to verify the (1) efficacy of physical activity in preventing and treating influenza in humans; (2) effectiveness of different physical activity programs in preventing influenza infection; (3) effects of physical activity at different stages of influenza illness; and (4) adoption of physical activity to the population for preventing influenza by gradually develop physical activity as a practical tool for influenza prevention and treatment.

### Elucidate the Physiological Mechanisms Through Which Physical Activity Helps Combat Influenza

Researchers have assessed symptoms, respiratory virus levels, and number of immune cells in body fluids to understand the role of physical activity in influenza prevention and control. This method of extrapolation based on phenomena can only evaluate the positive or negative effects of physical activity on influenza prevention. It is not yet possible to explain the physiological mechanisms through which physical activity affects the body’s resistance to influenza viruses, reveal the physiological mobilization characteristics of physical activity against influenza, and determine whether the anti-influenza effect of physical activity is to contain the infection or attenuate the virus. Therefore, we have not constructed the anti-influenza programs with physical activity, and we have also not clarified the focus of physical activity for anti-influenza. To develop the anti-influenza function of physical activity, subsequent studies should focus on the physiological mechanisms through which physical activity prevents and controls influenza and determine the (1) mechanism of the broken window period of human immunity caused by physical activity and effective countermeasures; (2) mechanism through which physical activity enhances human immunity and resistance generation for influenza prevention; (3) physiological mechanism through which physical activity influences the course of influenza illness. These results can inform the development of physical activity as a controlled measure for influenza prevention by ensuring the safety and effectiveness of these methods.

### Explore the Relationship Between Physical Activity and Influenza Transmission

Although moderate physical activity strengthens the body’s ability to resist infection by the influenza virus, it does not directly kill the virus. Physical activity participants are also at risk of influenza infection and transmission; hence, encouraging people to practise physical activity against influenza requires confronting the relationship between physical activity and influenza transmission. The previous studies have only described the positive or negative tendencies of physical activity to combat influenza; while physical activity can reduce the damage of influenza in individuals, there is also a risk of worsening influenza infection in groups, and even the risk of physical activity participants becoming mediators of influenza transmission. Follow-up studies are needed to assess how physical activity can enhance human resistance to influenza virus infection by attenuating the infectiousness or reducing the number of influenza viruses. If physical activity can effectively attenuate the infectiousness of influenza viruses, then people should be generally mobilized to participate in physical activity during the influenza season, which is tantamount to establishing a herd immunity mechanism to mitigate the harm of influenza virus transmission. If physical activity only reduces the amount of influenza virus in the patient’s body but does not reduce the infectivity of influenza virus, then physical activity is only beneficial to the participant and healthy people should take good personal protection to physically block transmission of the influenza virus. If physical activity is accompanied by exhalation of influenza viruses, thus strengthening virus transmission, then residents should be advised to reduce physical activity to control the transmission channels during the influenza season (does participation in physical activity help to transmit the influenza virus?) and avoid the emergence of more infectious influenza viruses (do physical activity participants serve as a good petri dish for influenza virus evolution?).

### Develop Exercise Prescriptions to Prevent and Treat Influenza

Moderate physical activity will work along several lines to prevent influenza by strengthening the resistance and immunity of the body, relieving anxiety, and improving sleep quality to ensure that our bodies are in good condition to resist influenza virus infection [11, 30]. Although researchers have encouraged people to participate in physical activity to prevent influenza, none have yet explicitly reported an appropriate physical activity regimen. Only a few programs have been published in the media, e.g. Webber, Yun and Whitfield recommended at least 150 min/week of moderate intensity aerobic exercise (e.g., brisk/speed walking, swimming, running, and stair climbing) [14]. Although it is recognised that physical activity may help prevent and treat influenza [40], and has positive effects on both the prevention and prompt treatment of early influenza in humans, it is not yet clear exactly how to put this into practice. Only a portion of subjects benefit from physical activity in terms of influenza prevention, alleviating the patients’ symptoms and increasing the recovery efficiency [41]; therefore, the ability of physical activity to improve the body’s resistance to infection by the influenza virus may vary among individuals (e.g., physical function, the environment of infection, physical activity regimen). Once physical activity is used as a preventive measure against influenza, it needs to be as reliable as medical treatment. However, no scientific evidence exists to support a corresponding exercise prescription, and we are limited to recognising that moderate physical activity can help prevent and treat influenza infections without specific operational tools (e.g., physical activity regimens, monitoring physiological indices, and dynamic management). Future studies must emphasize exercise prescriptions (1) for influenza prevention; (2) for the early stage of infection; (3) for the healing and recovery period; and (4) with subpopulation applicability, to promote the translation of research findings on the effects of physical activity in preventing and treating influenza from theory to application.

## Conclusion

It is important to avoid the risk of infection by not only influenza viruses but also by other infectious agents during the broken window of immunity caused by high-intensity endurance physical activity, even though evidence has shown benefits of high-intensity physical activity in improving physical fitness. Based on the practical need for physical activity for influenza prevention and control, subsequent studies should increase evidence from clinical human trials (strictly guaranteeing the human health safety in physical activity interventions) to test the effectiveness of physical activity to improve human ability in preventing and controlling influenza, reveal the physiological mechanisms and role of exercise participants in influenza transmission, and develop exercise prescriptions for influenza prevention and control to promote the translation of relevant research findings from theory to application. This will also support physical activity as a practical tool for influenza prevention and treatment.

## Data Availability

Not applicable.

## Abbreviations

COVID-19: Corona Virus Disease 2019
WHO: World Health Organization
IL-6: Interleukin 6
HAU: Hemagglutinating units
IgAs: Immunoglobulin A in saliva
Albs: Albumin in salivary
CON: Sedentary control
MOD: Moderate
PRO: Prolonged
IFN-γ: Interferon-γ
Th1: Type 1 T-helper
Th2: Type 2 T-helper
